# Structure of isolated Z-disks from honeybee flight muscle

**DOI:** 10.1007/s10974-017-9477-5

**Published:** 2017-07-21

**Authors:** Mara Rusu, Zhongjun Hu, Kenneth A. Taylor, John Trinick

**Affiliations:** 1 Astbury Center, University of Leeds, Leeds, LS2 9JT UK; 2 Institute of Molecular Biophysics, Florida State University, Tallahassee, FL 32306-4380 USA

**Keywords:** Cryo electron tomography, Subtomogram averaging, Z-disk, Indirect flight muscle, α-actinin

## Abstract

The Z-disk is a complex structure comprising some 40 proteins that are involved in the transmission of force developed during muscle contraction and in important signalling pathways that govern muscle homeostasis. In the Z-disk the ends of antiparallel thin filaments from adjacent sarcomeres are crosslinked by α-actinin. The structure of the Z-disk lattice varies greatly throughout the animal kingdom. In vertebrates the thin filaments form a tetragonal lattice, whereas invertebrate flight muscle has a hexagonal lattice. The width of the Z-disk varies considerably and correlates with the number of α-actinin bridges. A detailed description at a high resolution of the Z-disk lattice is needed in order to better understand muscle function and disease. The molecular architecture of the Z-disk lattice in honeybee (*Apis mellifera*) is known from plastic embedded thin sections to a resolution of 7 nm, which is not sufficient to dock component protein crystal structures. It has been shown that sectioning is a damaging process that leads to the loss of finer details present in biological specimens. However, the *Apis* Z-disk is a thin structure (120 nm) suitable for cryo EM. We have isolated intact honeybee Z-disks from indirect flight muscle, thus obviating the need of plastic sectioning. We have employed cryo electron tomography and image processing to investigate the arrangement of proteins within the hexagonal lattice of the *Apis* Z-disk. The resolution obtained, ~6 nm, was probably limited by damage caused by the harshness of the conditions used to extract the myofibrils and isolate the Z-disks.

**Electronic supplementary material:**

The online version of this article (doi:10.1007/s10974-017-9477-5) contains supplementary material, which is available to authorized users.

## Introduction

The Z-disk (also known as Z-band or Z-line) forms the boundaries of the sarcomeres, the contractile units of striated muscle. In the Z-disk, the ends of thin filaments of opposing polarity from adjacent sarcomeres interdigitate and are crosslinked by α-actinin bridges (Z-bridges) (Luther 2009). This end of thin filaments is stabilized in the Z-disk by the actin capping protein CapZ (Narita et al. 2006). Thin filaments extend from the Z-line toward the middle of the sarcomere and overlap in the A-band with thick filaments consisting of myosin II and accessory proteins aligned in register at the M-line (Lange et al. 2005; Schoenauer et al. 2005).

α-actinin is a member of the spectrin family of actin binding proteins. The molecule consists of an N-terminal actin binding domain which is connected via a flexible neck region to a variable number of spectrin repeats, depending on the organism. The C-terminal end consists of a calmodulin like domain. In the Z-bridges α-actinin exists as a rod shaped antiparallel homodimer (200 kDa). Recently the crystal structure of the homodimer in its closed conformation was solved. It has been shown that upon binding of titin Z repeat 7 in the Z-disk, in the presence of PIP2, the molecule can adopt an open conformation that favours actin binding (Ribeiro et al. 2014). Electron microscopy of filamentous actin and α-actinin rafts has shown that the molecule is able to bind antiparallel actin filaments as well as parallel ones at various angles. α-actinin was also shown to attach itself actin domains in the same filament, indicating that α-actinin is a flexible linker, rather than a rigid spacer (Hampton et al. 2007).

Within the Z-disk there are over 40 proteins that create an extensive network of interactions. Some of the components have mainly a structural role (e.g. CapZ, α-actinin, telethonin), but several of them have been shown to be able to translocate to the nucleus or sarcolemma (e.g. podin family members). Within the nucleus some of these proteins act as regulators for gene expression that ultimately dictate muscle growth, development and wasting. Excellent reviews detailing known interactions within the lattice as well as involvement of Z-disk proteins in various signalling pathways are available (Frank et al. 2006; Frank and Frey 2011). Recent studies focusing on Z-disk components indicate a highly dynamic Z-disk that is involved in a multitude of signalling pathways that govern muscle homeostasis and stretch sensing (Gautel 2011). There is thus a need to correlate biochemical and biophysical data providing insight into how muscle develops and ages with high resolution structural data that will allow accurate docking of known protein crystal structures within the Z-disk lattice.

While vertebrate and insect Z-disks perform similar functions in striated muscle there are some distinctions between the two muscle types. The width of the Z-disk varies in vertebrates with muscle type, with fast twitch muscles narrower (30–50 nm) than slow twitch and cardiac muscle (100–140 nm). The width correlates closely with the number of α-actinin cross bridges present (Luther 2000; Luther et al. 2002). Thin filaments end in a tetragonal lattice that has two distinct appearances, depending on the state of the muscle. In relaxed muscle the lattice has a small square appearance, while in active muscles a basket weave lattice is seen (Burgoyne et al. 2015; Perz-Edwards and Reedy 2011).

In insects, depending on the correlation between nerve input and wing beat, two types of flight muscle can be distinguished: synchronous and asynchronous. In the case of synchronous flight each contraction is initiated by nerve input, whereas as in asynchronous flight muscle the frequency of contraction is higher than that of the nerve impulses. Asynchronous indirect flight muscles (IFM), such as those found in honeybee, are characterized by long range crystal-like arrangement of the myofilaments (Iwamoto et al. 2006). The giant vertebrate protein titin is not expressed in IFM, but related protein classes with similar functions have been identified: Sls (*Drosophila sallimus*) and projectin/twitchins. Kettin (540 kDa), one of the Sls gene products, is the predominant isoform in IFM. Kettin binds to actin and α-actinin and is crucial for thin filament stability and for maintaining passive tension within the myofibril (Kulke et al. 2001). A distinctive hexagonal lattice characterizes the lattice of the Z-disk in IFM. The three dimensional (3D) structure has been resolved to a resolution of 7 nm from plastic embedded and sectioned *Apis* flight muscle. The specimens were imaged at different tilt angles (at 0°, 45° and 90°) and crystallographic methods were employed for volume reconstruction. The honeybee Z-disk is 120 nm thick, with the thin filament overlap in the central region being around 80 nm. The reported symmetry was p312 with the threefold axes parallel to the myofibril axis and the twofold axes located in the transverse central plane, perpendicular to the myofibril axis (Cheng and Deatherage 1989). 3D image data from tilted specimens was combined with structural data from oblique sections through the Z-disk to determine the orientation of thin filaments within the lattice as well as the relationship with thick filaments in the A-band (Deatherage et al. 1989). Within the 3D volume connecting densities between antiparallel and parallel thin filaments were resolved, but due to the relatively low resolution (7 nm) the density map lacks high-resolution features, thus crystal structures of the Z-disk components could not be docked accurately.

The Z-disk is mechanically strong and able to withstand forces developed during sarcomere contraction. Studies focusing on the chemical stability of the Z-disk date from 1962 when Garamvölgy and coworkers showed that all sarcomere components in honeybee myofibrils exposed to mild acids and high salt solutions solubilize, except for in the Z-disk (Garamvolgyi et al. 1962). In 1974 a seminal report on Z-disks isolated using lactic acid came to the remarkable conclusion that the Z-disk hexagonal lattice withstood treatment with lactic acid (Saide and Ullrick 1974). However, the Fourier transform (FT) of lactic acid isolated Z-disks showed one order of diffraction, which indicates that high resolution features are not preserved. The diameter of the Z-disks also varied depending on the buffer. In lactic acid the Z-disks had a diameter of 3 μm (Saide and Ullrick 1974), whereas the physiological diameter is around 2.4 μm (Deatherage et al. 1989). If the isolated Z-disks were transferred into a physiological buffer the diameter of the Z-disks decreased to what is typically observed in intact myofibrils.

Plastic sectioning is a powerful technique that has been employed for decades to study muscle ultrastructure. Resin embedded muscle diffracts to a resolution of 0.8 nm, whereas in sectioned muscle the resolution is 5 nm at best (Sader et al. 2007). These data therefore indicate that the cutting process damages the structure of biological specimens. The Z-disk is a thin structure (120 nm) and as demonstrated by Saide and Ullrick can be successfully isolated using biochemical methods (Saide and Ullrick 1974), thus there is no need to section muscle in order to study the Z-disk structure. The aim of our work was to determine methods of isolating well preserved Z-disks and to establish protocols for plunge freezing and cryo electron microscopy and tomography in order to investigate the molecular architecture of the hexagonal Z-disk lattice. Modern image processing techniques, such as subtomogram averaging, could then be utilized to study the 3D structure of the Z-disk. Subtomogram averaging is a powerful technique and was recently employed to study the architecture of cardiac Z-disks in plastic sections (Burgoyne et al. 2015).

Here we report on the first attempt to reconstruct the isolated Z-disk in 3D using cryo-electron tomography (cryoET). We use Z-disks from the honeybee *Apis mellifera* flight muscle. The 3D model shows considerable detail and suggests how this approach may be improved to facilitate the ultimate goal of a molecular description of the Z-disk from different striated muscle sources. The resolution achieved, ~6 nm, was too low to accurately dock component crystal structures, almost certainly due to the relatively harsh extraction conditions used to isolate the Z-disks, but if gentler conditions could be found substantially better resolution and preservation should be attainable.

## Results

### Isolation and characterization of *Apis* flight muscle Z-discs

**Fig. 1:**
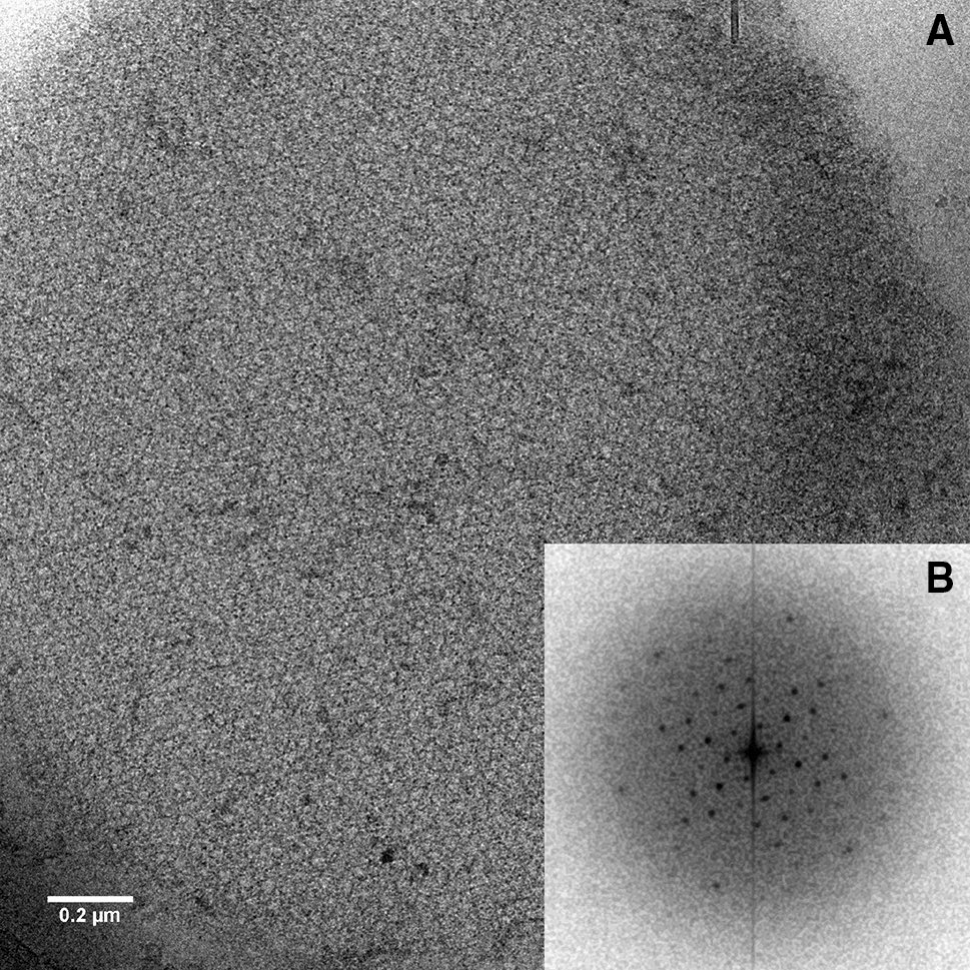
Preparation of Z-disks. **a** Cryo-EM micrograph of a Z-disk. **b** Fourier transform of **a**

Z-disks were isolated from honeybee indirect flight muscle. After dissection of the muscle from the thorax it was homogenized into myofibrils, which were separated from soluble components by repeated cycles of gentle centrifugation and resuspension in physiological ionic strength buffer. The washed myofibrils where either used immediately or stored at −80 °C in glycerol. The Z-disks were prepared by extracting the myofibrils with a high ionic strength solution (0.7 M KCl, 0.6 M KI, 0.08 M NaHCO_3_ pH 8). The extraction process was monitored using phase contrast light microscopy and negative stain EM. Figure 1a, b show a cryo-EM micrograph of a Z-disc and its Fourier transform, which extends to six orders, i.e. to about 8 nm.

### Tomogram of frozen hydrated Z-discs

**Fig. 2:**
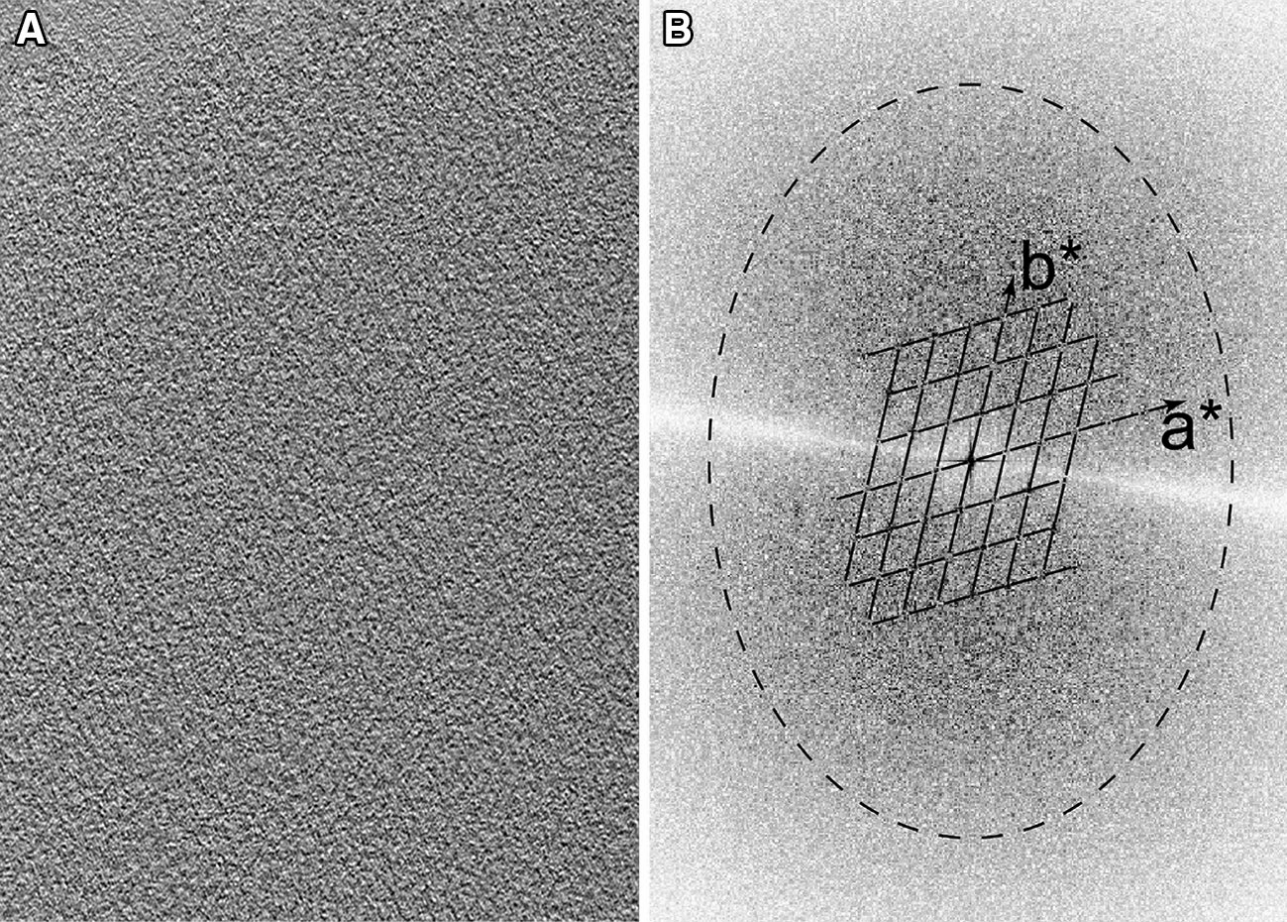
Tomogram and its Fourier transform. **a** A section through the center of the tomogram showing the region used for the subvolume averaging. The *small triangular patch at the upper left* does not contain Z-disk. At this magnification, the lattice can be seen but details are too noisy to be distinguished. **b** The central section of the Fourier transform of the tomogram in which the peaks in the reciprocal lattice can be visualized. The reciprocal lattice is drawn out. The *faint white line* running horizontally across the transform shows the orientation of the tilt azimuth with respect to the tomogram. *Dashed elliptical line* marks the 6 nm resolution limit

**Fig. 3:**
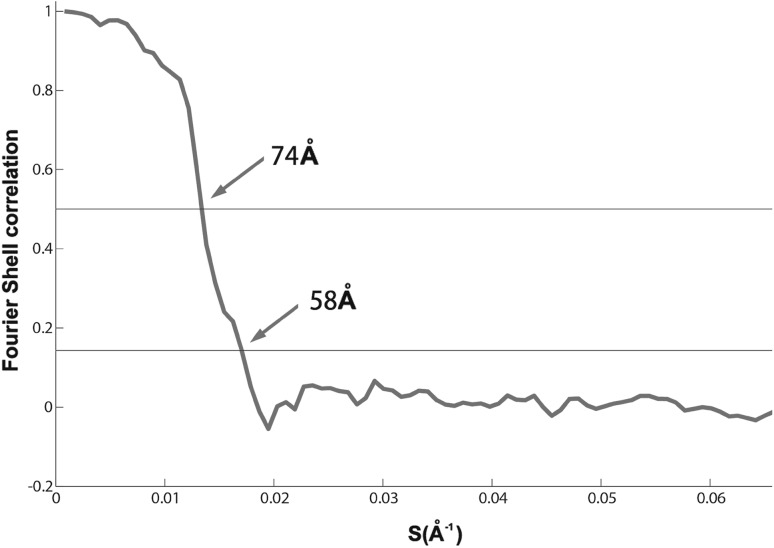
Resolution estimation from Fourier shell correlation. The *plot* indicates a resolution of about 6 nm using the 0.143 standard cut-off

The 2-D repeating motif in the low pass filtered tomogram is barely visible (Fig. 2a). However, a repeating lattice is demonstrable in the Fourier transform of the tomogram (Fig. 2b). After subvolume extraction, symmetrisation and averaging, we obtained a final density map with resolution about 6 nm (Fig. 3). Our resolution estimate is reasonable given the fact that there are no actin subunits visible in the global average reconstruction. Had they been resolved, a resolution estimate of 5.5 nm, the axial spacing between subunits, would have been reasonable. The relatively low resolution of the map almost certainly results from damage to the Z-disks caused by the harshness of the initial extraction solution, which was high salt including high potassium chloride and iodide. The low resolution probably indicates partial denaturation and likely loss of components.

**Fig. 4:**
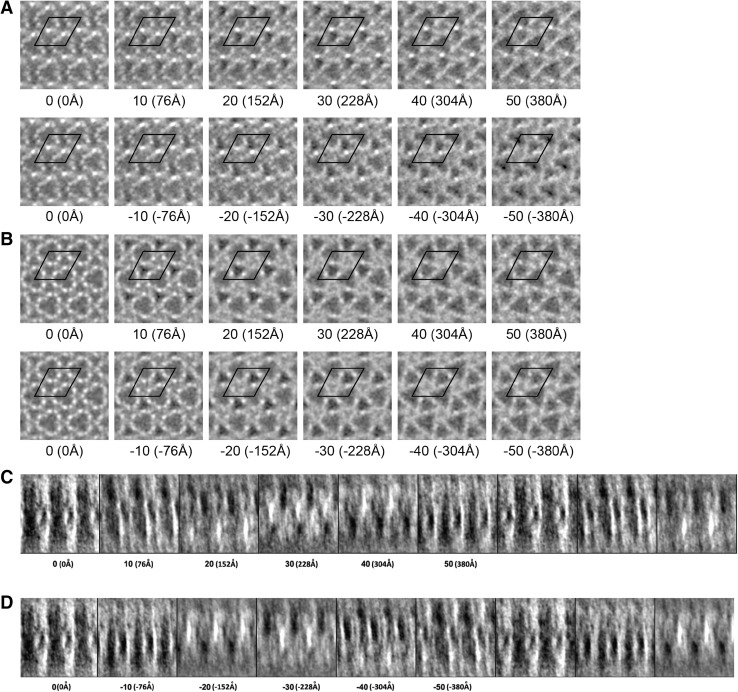
Selected sections through the averaged Z-disk subvolumes. Note that *white* represents protein in this display. **a** These are sections through the raw tomogram computed from the first 67 images prior to “symmetrisation”. *Numbers* give the actual section in the reconstruction. *Parenthetical numbers* are the actual *z* coordinate. *Black lines* mark the unit cell. The most characteristic feature is the large *triangular shaped channel* located at the lattice positions in every section, though its *triangular shape* varies when passing through the Z-disk. **b** Sections through the symmetrized reconstruction. The improvement in signal-to-noise ratio is considerable despite using only the first half of the tilt series. In the centre of the tomogram, section 0, there are two groupings of three *white spots*, which represent actin filaments coming from opposite sides of the Z-disk. Note that the average density in the centre of the *triangular shaped channels* changes with distance along the *Z-axis* suggesting the presence of features not yet recognizable or identifiable. **c** and **d** are side views before and after symmetrisation

We can identify the position of actin filaments from the density map where they appear as small but intense white densities (Fig. 4a). The plausible actin filaments are not symmetrical in density due to the missing wedge of data in Fourier space caused by limitation of the maximum tilt angle to about 70°. The actin filaments are more clearly defined after imposing symmetry on the subvolume average (Fig. 4b). Every two corresponding sections, for instance, section −50 and section +50, are symmetrical. Figure 4c, d shows the side view and effect of symmetrisation.

**Fig. 5:**
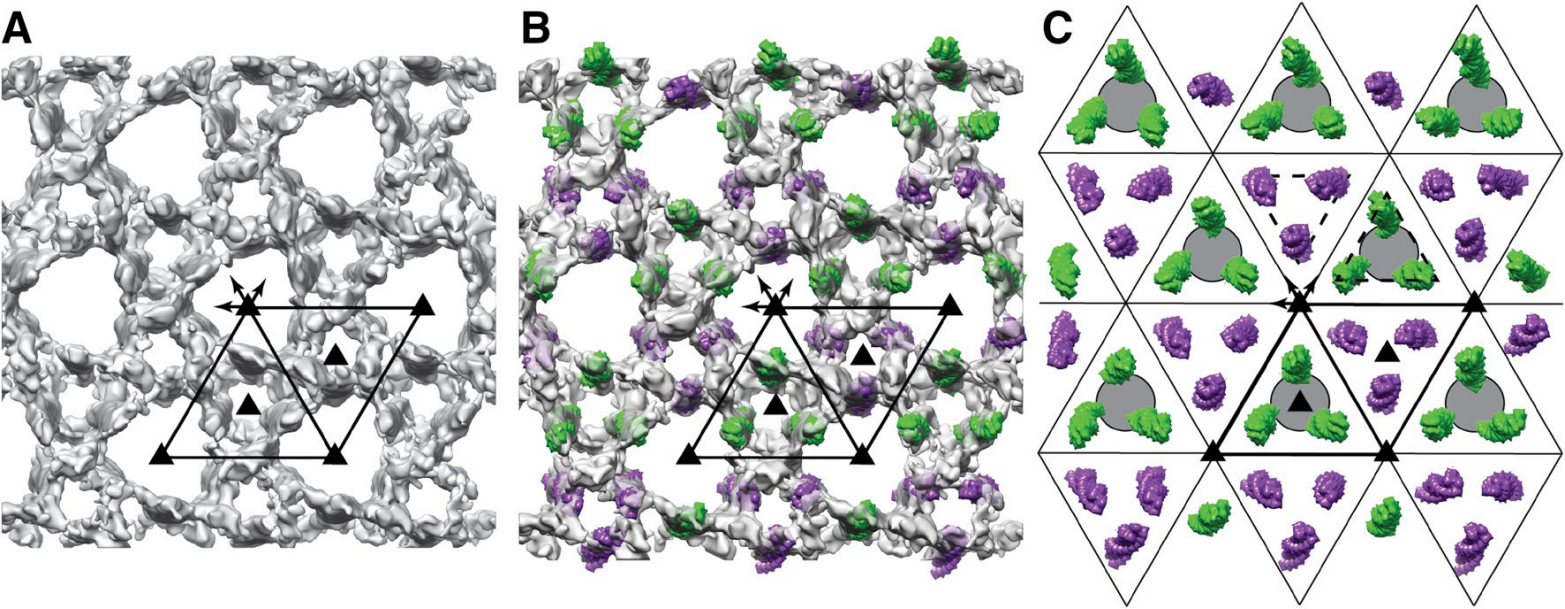
Symmetrized reconstruction viewed as a surface. **a** Subvolume average alone with the unit cell marked. The lattice positions, corners of the unit cell, are located in the centre of the large triangular channel. Smaller “channels” bounded on each corner by an actin filament occupy the trigonal positions within the unit cell. *Arrows* indicate the position of in-plane twofold rotation axes, all of which are aligned to the unit cell edges in the center of the Z-disk. A family of screw axes required in the 2-sided plane group p321 are not shown but run parallel to the cell edges passing through the midpoints. **b** F-actin density maps computed from an atomic model are superimposed on the same density map as shown in **a**. *Green* actin filaments converge at the backside of the Z-disk and diverge as they approach the observer. *Violet* actin filaments are oriented just the opposite. **c** Only the F-actin density maps computed from an atomic model are shown here. The *gray disc* at the back of the *green* F-actin trimers represents the position of a thick filament in the sarcomere at the back. The *violet* F-actins extend downward toward the gray thick filaments ending at a position midway between thick filament pairs. Each *violet* trimer contributes a pair of actin filaments to each thick filament but each actin filament is shared between a pair of thick filaments. The slight angle that the thin filaments have in the Z-disk enables them to be positioned at the diad positions of the A-band lattice while converging toward a trigonal position in the Z-disk lattice. The *dashed triangles in the upper left* show how filaments from each of the two groups of F-actin trimers surrounding the triangular channel on the lattice positions contribute to the apparent rotation of the channel as it traverses the Z-disk

**Fig. 6:**
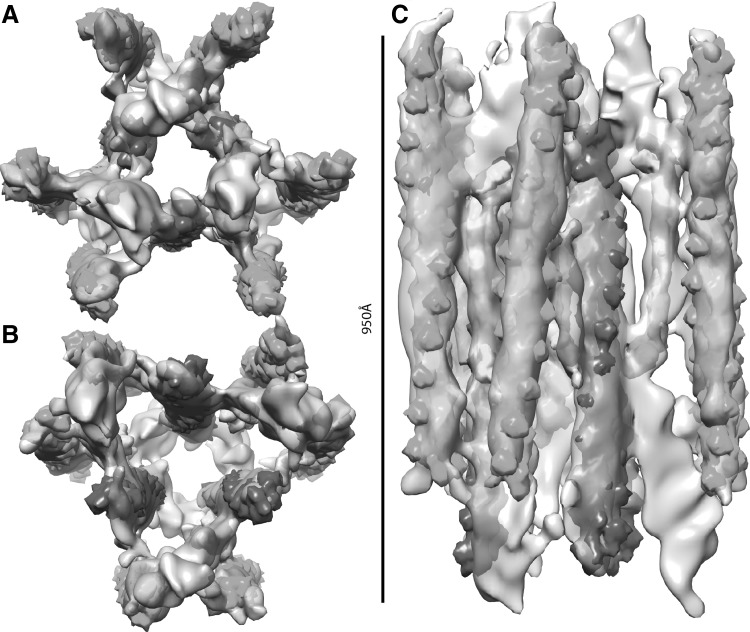
Actin trimers and cross-links. Density maps representing actin filament trimers are *coloured green* and *violet*. A plausible α-actinin cross-link is shown in *gold*. **a, b** Images show views through actin filament trimers from opposite sides of the Z-disk. In **a** the *violet* F-actins converge from the *bottom* toward the observer at the *top*, and in **b** they diverge from the *bottom* toward the observer at the *top*. **c** Longitudinal view of the region shown in **a, b**. Density connecting both parallel and antiparallel actin filaments is found in the subvolume average. Only one of these appears to be of a size and shape that could accommodate an α-actinin molecule and it occurs between antiparallel actin filaments. The density is significant only for one antiparallel cross-link. The density corresponding to the other cross-links required by the symmetry is weaker and not as prominent at the contour level displayed. Note that the F-actin and α-actinin density maps are not “fit” to the reconstruction, but merely placed manually to aid interpretation. Quantitative fitting would be meaningless unless the F-actin subunits had been resolved

Figure 5 shows the model in surface representation, filtered to 6 nm resolution. The 3D density map is very similar to the previous report obtained by crystallographic (spatial) averaging of a tilt series collected from a Z-disk in a stained and embedded plastic section (Cheng and Deatherage 1989). We note that the 2-sided plane group reported, p312, is apparently a misprint and should be p321. In p321 the twofold axes in the plane of the Z-disc run parallel to the unit cell edges where they relate F-actins extending to opposite sides (Fig. 6), whereas in p312 the twofolds lie perpendicular to the unit cell edges. The earlier structure shows clearly that the twofolds lie parallel to the unit cell edges.

Triangular shaped density groupings are visible in cross sections through the average of all subvolumes, especially in sections −50 and +50 (Fig. 4a) but they are much more obvious following symmetrisation (Fig. 4b). In the centre of the Z-disk, a large solvent channel is defined by the presence of six actin filaments of alternating polarity, which give the channel the appearance of a triangle with rounded edges. As the channel progresses out from the centre, one group of parallel filaments diverges while the other group converge so that at a distance of 30 nm from the Z-disk centre, a triangle with sharp corners is observed with the diverging filaments defining the edges and the converging filaments defining the corners. Moving the other direction to −30 nm, converging and diverging filament groups change roles with the consequence that the triangular profile rotates. In agreement with a previous report (Saide and Ullrick 1973), the triangle shape rotates smoothly as it passes through the Z-disk, while passing through a more rounded intermediate near the centre of the Z-disk. If a triangle is drawn to fit the channel profile in section −50, it rotates ~22° by the time it reaches section 50. Cheng and Deatherage (1989) observed a rotation of 11° for each trimer lining the small channel. Referred to the large channel, this produces a rotation of 22°, exactly what is observed here (Fig. 4).

Each unit cell (bold black line) includes six actin filaments organized into two groups of three centred on trigonal positions of the unit cell. Three are coloured violet and three are coloured green in Fig. 5. The three actin filaments of the violet group (Fig. 5b, c) are converging in the front and diverging towards the back, while the three actin filaments of the green group are converging at the back while extending and diverging toward the front. The length of the bold lines defining the unit cell edges is 52 nm, which is the distance between two myosin filaments in the flight muscle lattice (Millman 1998). Myosin filaments would be exactly located at the centre of three converging actin filaments in the x–y plane.

Neighbouring green and violet actin filaments are antiparallel to each other. Connecting them is a density that is long enough to accommodate α-actinin (Fig. 6, gold). We assign this density to α-actinin because the density is the correct length while being positioned to one side of the antiparallel actin filaments as predicted in several models (Liu et al. 2004; Ribeiro et al. 2014). Besides F-actin and α-actinin, there is additional density assignable neither to α-actinin nor F-actin. We suggest this density may be part of kettin (Fig. 6c), because kettin connects the end of the myosin filament to the actin filament and interacts with α-actinin (Hampton et al. 2007; Ribeiro et al. 2014; Vigoreaux 1994). CapZ is at the plus end of actin filament, which in this model is the converging end of the densities assigned to F-actin. Due to its small size, CapZ cannot be identified at the current resolution.

Despite the fact that we symmetrized the reconstruction, we do not see density sufficiently long to accommodate α-actinin at every location where antiparallel F-actins are close enough to form a cross-link. Of the six green F-actins surrounding the three violet F-actins in Fig. 6, only three have plausible α-actinin cross-links. The uncross-linked F-actins are bound indirectly to the inner three through parallel F-actin cross-links.

## Discussion

The major advantage of studying the Z-disc in its isolated form is that it is thin enough not to necessitate sectioning. Its composition can also be readily monitored. Studies on the structure of the Z-disk go back as far as 1962 (Garamvolgyi et al. 1962), while attempts to isolate Z-disks for biochemical characterization appear about a decade later (Saide and Ullrick 1974). However, almost all structural reports of Z-disk structure utilized plastic sections of fixed and embedded muscle tissue. One other route not yet attempted to producing a thin section or lamella is focussed ion been milling (FIB) where the sample is abraided and milled to a thin lamella by a beam of ions, but this has not yet been tried with muscle specimens.

### Comparison with previous results

One conspicuous feature appearing consistently in all the Z-disk structures reported, including the present results, is the presence of pronounced triangular shaped channels extending through the entire Z-disk, apparently unobstructed by other features (Cheng and Deatherage 1989; Saide and Ullrick 1973). One large channel is located on the lattice positions while two smaller channels are found at the trigonal positions of the unit cell. The large solvent channel passes through the entire Z-disk with only minor fluctuations in density. However, the density within the large channel appears higher around 15 nm from the Z-disk centre and falls off after that (Fig. 4b). The small solvent channels do not continue through the entire Z-disk but start to fill with density, presumably cross-links, as they converge, becoming noticeably congested at 7.5 nm and completely filled at 23–30 nm from the centre. We think this feature of our reconstruction corresponds to the C4 connections of Cheng and Deatherage (1989). The four other connecting densities, C1–C3 and C5 are less easy to identify because actual coordinates were not given in the earlier work and our reconstruction is too noisy to define them.

The large solvent channel has the interesting feature that it appears to rotate as it passes through the Z-disk (Deatherage et al. 1989; Saide and Ullrick 1973). This rotation is a consequence of the fact that the F-actins within the Z-disk are not oriented parallel to the fibre axis. Instead they are angled so that on one side they converge toward a point that lies immediately above a thick filament from the adjacent sarcomere. As they diverge, they come to lie at the diad positions between thick filaments from the sarcomere on the opposite side (Fig. 5c).

## Future prospects

One of the advantages of working with isolated Z-disks is the relatively more focussed analysis possible using a sarcomeric component rather than a whole sarcomere. The possibility of imaging in 3-D the native structure rather than a fixed and stained representation is the second big advantage. With isolated Z-disks, we can envision “decorating” the structure with recombinant signalling molecules, possibly engineered to have labelling sites for heavy atom clusters. The isolated Z-disk would provide an example of a relaxed or tension free structure, which would make it ideal for decorating with molecules that under relaxed conditions migrate to the Z-disk.

The disadvantage of working with isolated Z-discs is the harshness of the conditions currently used to prepare them. However, if milder conditions can be found resolution could in principle be much higher, potentially sub-nm, for instance taking advantage of the repetitive structure of the Z-disc to allow sub-tomogram averaging of whole unit cells but also individual components such as F-actin and α-actinin. At such higher resolution, localization and docking of the many Z-disc components would become possible. Milder conditions may be more feasible using vertebrate muscle, since methods are known for the selective cleavage of I-band titin (Higuchi 1992) and depolymerisation of thin filaments outside the Z-disk by gelsolin (Funatsu et al. 1990), which are the two main conditions that have to be met to liberate Z-disks.

## Materials and methods

### Sample preparation

Indirect flight muscle was harvested from the thorax of *Apis mellifera* obtained from a local bee keeper. Myofibrils in suspension were prepared according to previously published methods (Bullard et al. 1973; Saide and Ullrick 1974) with the following modifications. The IFM were collected in ice cold sucrose containing buffer (0.3 M sucrose, 0.1 M KCl, 0.01 M potassium phosphate pH 7, 1 mM MgCl2, 2 mM EGTA, 0.02 M NaN_3_ and EDTA-free Protease Inhibitor Cocktail Tablets) followed by homogenization using a Wheaton tissue grinder. Soluble proteins and sucrose were washed away by centrifugation in 0.1 M KCl, 0.01 M potassium phosphate pH 7 buffer. Myofibrils in suspension were stored in 75% glycerol at −80 °C. Intact Z-disks were isolated by incubating myofibrils on ice in a high ionic strength extraction solution containing 0.7 M KCl, 0.6 M KI, 0.08 M NaHCO_3_ pH 8 for 60 min. Following extraction Z-disks were either negatively stained or plunge frozen for further electron microscopic investigation.

Carbon coated grids were made hydrophilic by glow discharging for 40 s at a high tension of 10 kV in a Cressington 208 Carbon Coater. After the Z-disk suspension was added to the grid it was left to settle for 10–15 s followed by washing with low salt buffer (25 mM HEPES, 100 mM NaCl). The washing step is crucial for the removal of salts present in the extraction buffer, ensuring that both negatively stained and plunge frozen grids were free of contamination. The stain of choice was 1% ammonium molybdate pH 7. Plunge freezing was carried out using Quantifoil grids (Agar Scientific) in a Vitrobot Mark IV (FEI) at 4–5 °C, 90–100% humidity using a blotting force of 3–5 for 4–6 s.

### Electron microscopy and tomography

Electron microscopy of stained and frozen grids was carried out using a FEI Tecnai G2 Spirit and photographed using a 2k × 2k Gatan CCD camera under low dose conditions. Tilt series were recorded at the Medical Research Council’s Laboratory for Molecular Biology (LMB) in Cambridge on the FEI Titan Krios electron microscope equipped with the FEI Falcon II Direct Electron Detector. Magnification was 22,500×; pixel size was 3.8 Å. The raw tomogram was computed with a binning factor of 2 for an effective pixel size of 7.6 Å. Tilt series were recorded using the Saxton acquisition scheme (Saxton et al. 1984). Tilt angles ranged from −69.87° to +67.66° starting at 0°, 96 total images, with an initial step of 2°. The average electron dose/micrograph was ~0.7 electrons/Å^2^. A total of 12 tilt series, designated tomo1–tomo12, were collected but only one proved to be useful, tomo9. The others had poor resolution due to either low intrinsic order, excessive extraction of actin or other structural Z-disk proteins, or due to excessive radiation damage.

### Tilt series alignment

The tilt series were aligned using marker-free alignment and the tomogram computed by weighted back projection using the PROTOMO software package (Winkler and Taylor 2006). Generally, PROTOMO uses a cross correlation between the tilt series image and a reference calculated by reprojecting a preliminary back projection image computed from all the previously merged tilt series images. This procedure prevents propagation of alignment errors in the low tilt angle images into the high tilt angle images. In addition, PROTOMO utilizes an area-matching algorithm in which the raw image is distorted according to a 2-D distortion matrix to match the reference image. Area matching utilizes the fact that the specimen is foreshortened according to the tilt angle and the tilt azimuth of the goniometer and this is reflected in its projection. Least squares fitting of the distortion matrices for the complete tilt series determines to what degree the specimen tilt azimuth and tilt angle differs from that recorded from the goniometer position. The correlation peak as well as the residuals from the least squares fit are recorded and output during area matching for examination by the user to determine the progress of the fitting.

During the tilt series alignment we found that correlation peaks disappeared after the 66th image (Supplemental Figure S1) due to radiation damage. The residuals from the area matching of the remaining images fell within 1% of ideal (Supplemental Figure S2). The tomogram used for the initial subvolume analysis was computed from the first 66 images which fell within an angular range of −69.88° to +44.88°. The final tomogram size was 600 × 864 × 160 voxels.

### Subvolume processing

Lattice positions were determined from a cross correlation map computed between the tomogram and a reference masked from within it. The Fourier transform was first filtered using a reciprocal lattice that contained three orders in a* and five orders in b*. Lattice positions were determined by peak fitting along a grid of predicted positions. Points on the predicted grid that fell outside of the actual Z-disc were removed as were any points that occurred too close to the edge of the tomogram. In total we extracted 399 subvolumes.

Since Z-disc could be considered a 2-D crystal, the initial Euler angle could be determined by the angular difference between horizontal axis of the tomogram and the direction of the ***a*** axis. The classification and alignment of motifs were performed using the I3 software package, which utilizes the scheme known as “alignment by classification” of class averages that themselves were obtained using multivariate data analysis and hierarchical ascendant classification (Winkler and Taylor 1999; Winkler et al. 2009).

The 3D map reconstructed from the whole tilt series was very noisy, because of the damaged second half tilt series and had an unacceptably large missing wedge. We adopted the following scheme to compensate for the diminished angular range and the higher damage suffered by the second half of the tilt series. After all the subvolumes had been aligned using alignment by classification, we utilized the symmetry of the crystalline lattice to generate all the symmetry related views of the Z-disk, thereby increasing the size of the subvolume data set by sixfold, and aligned them to the global average of the unsymmetrized tilt series. We then recomputed the tomogram using only the first half of the tilt series and using the alignment parameters of the expanded data set of subvolumes, which generated a new global average, which had a missing cone rather than a missing wedge and higher signal-to-noise ratio (Supplementary Figure S3). The resolution of final reconstruction is about 60 Å, determined through comparing the global average with an F-actin density map built from an F-actin atomic model as described below.

### Structural analysis

The final reconstruction was filtered to 60 Å resolution, according to above resolution estimation. The best contour threshold for observation is 1.15. The atomic model of the actin filament and α-actinin (PDB ID: 4D1E) (Ribeiro et al. 2014) were used to analyse Z-disc structure. The F-actin atomic model was made using the G-actin atomic structure (PDB ID: 2ZWH) (Oda et al. 2009) and the helical parameters appropriate to a 28/13 helix. There are 28 actin subunits in each F-actin. The density maps of F-actin and α-actinin were made using the PDB2MRC utility within the EMAN (Ludtke et al. 1999) package filtered to a resolution of 30 Å.

Density map and atomic models were displayed using UCSF CHIMERA (Pettersen et al. 2004).

### Resolution assessment

Coordinates for each raw subvolume were divided into two groups randomly. These subvolumes were divided prior to symmetry expansion. For each group the processing was the same as described for the subvolume averaging. A mask was generated that masked out everything but what is shown in Fig. 6a. The two reconstructions were then compared by FSC.

### Accession numbers

The reconstruction is in the EM database with the accession number EMD-8727. The tilt series image data is in the EMPIAR database with Accession Number EMPIAR-10095.

## Electronic supplementary material

Below is the link to the electronic supplementary material.

Supplementary material 1 (DOCX 938 KB)

## Abbreviations

IFM: Indirect flight muscle
FT: Fourier transform
