# Supplementary material for: Structure of isolated Z-disks from honeybee flight muscle
Source: J Muscle Res Cell Motil. 2017 Jul 21;38(2):241–50. doi: 10.1007/s10974-017-9477-5 (PMC5660141; doi:10.1007/s10974-017-9477-5)
Supplement: Supplementary file 1 — Supplementary material 1 (DOCX 938 KB) [file 10974_2017_9477_MOESM1_ESM.docx]

Supplemental Figures for Rusu et al., 2017


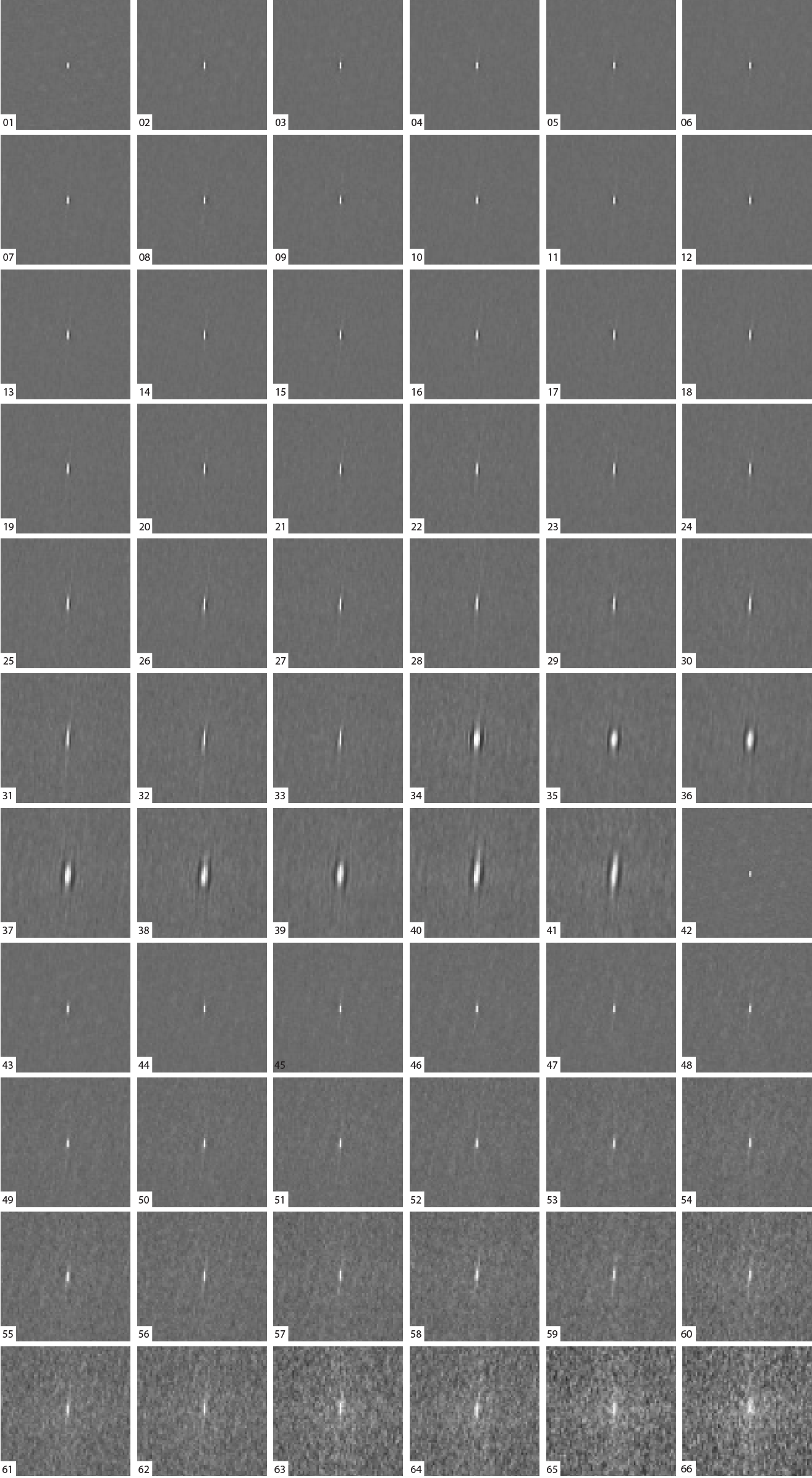


Figure S1. Correlation peaks for the final cycle of area matching in protomo. Image 00 (not shown) is the initial image of the tilt series and the first reference for the alignment. Images are numbered in the order recorded. The second half of the tilt series begins with image 42. The cross correlation peaks become significantly weaker after image 55, evidenced by the higher granularity of the background. Image 66 was the last usable image in the tilt series.


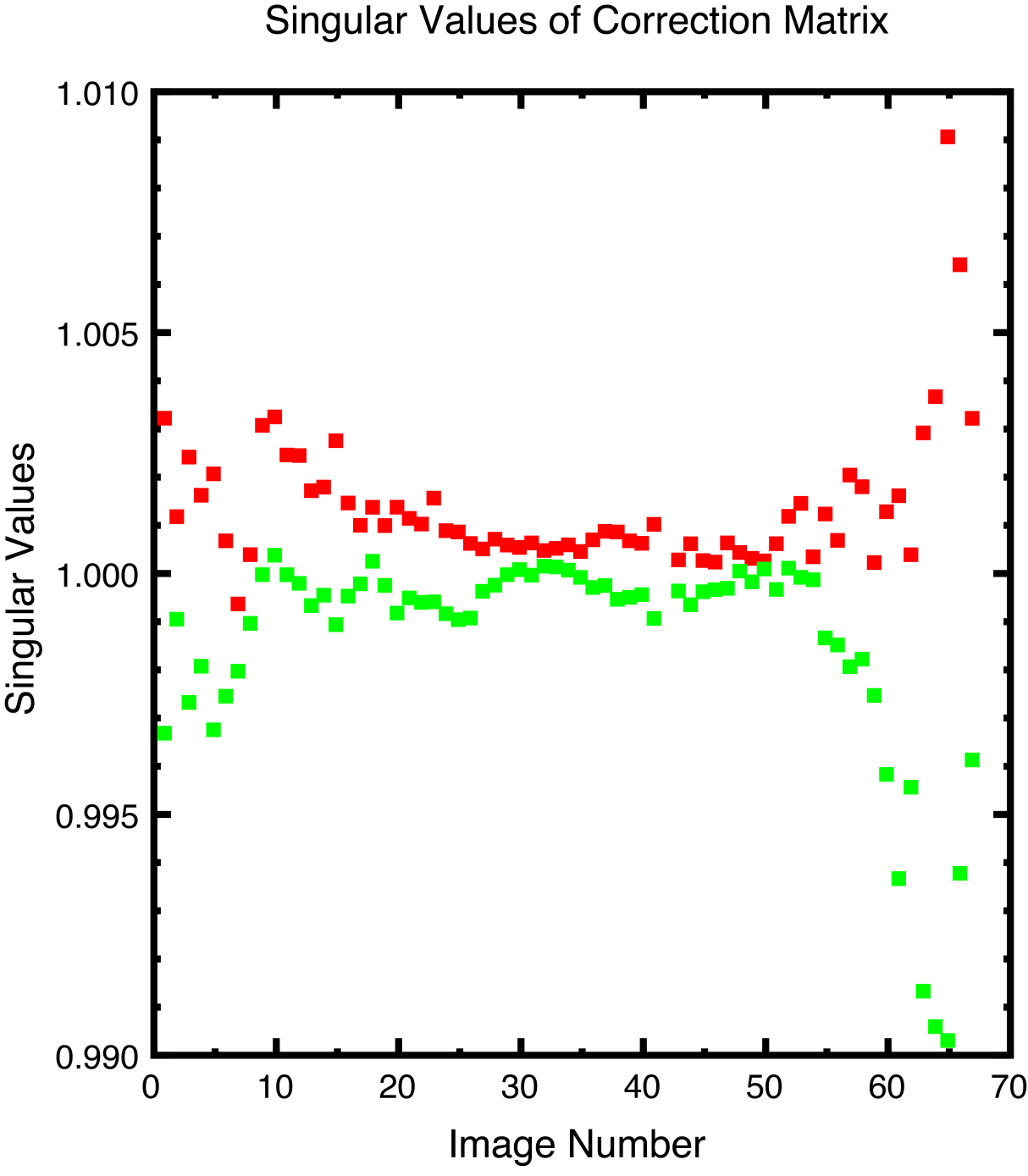


Figure S2. Singular values from the last cycle of area matching. The points represent how much of the image distortion determined by area matching cannot be explained by the two orientation matrices. Generally, images with acceptable fits have singular values that deviate from 1.0 by <0.01. Note that the deviations are larger for the last few acceptable images from the second half of the tilt series, whereas they are quite excellent even for the highest tilt angles of the first half of the tilt series. Image number 42 (not shown) is the image of the untilted specimen, which is actually the first image recorded and thus is never modified. Images 1-41 are the first half of the tilt series numbered in reverse order so that high angle tilts are at the sides and low angle tilts are in the middle. Images 43-66 are those images used from the second half of the tilt series in their normal order.


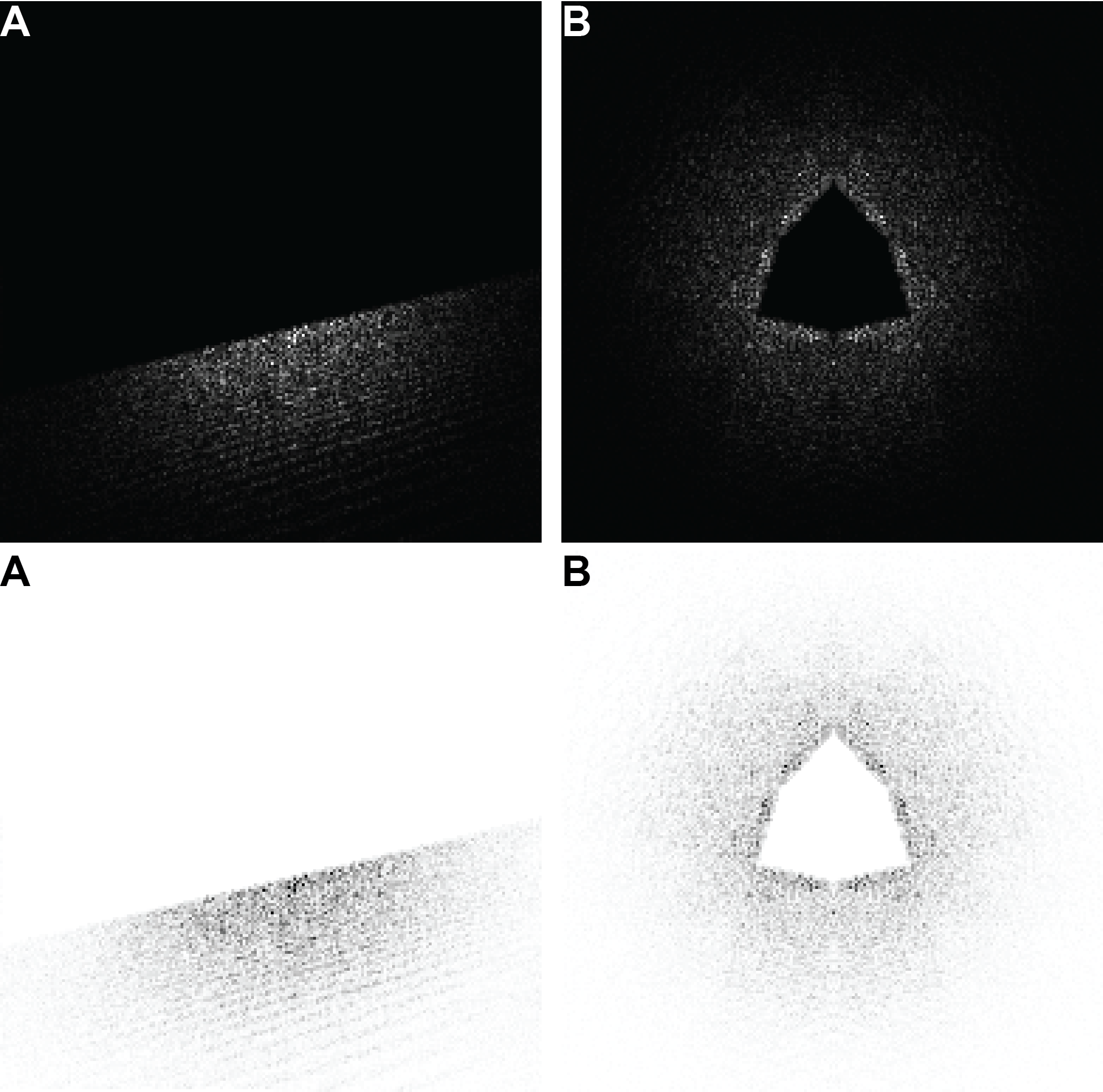


Figure S3. Missing wedge and cone. (A) A section from the Fourier transform of the global average from the tomogram computed with only the first half of the tilt series. This is not a central section from the transform, but rather an upper level plane chosen to emphasize both the higher resolution spots and how much data were eliminated when using only the first half of the tilt series. (B) Same upper level section but this time after applying the symmetrizing operations to the raw subvolumes prior to averaging. The vast missing wedge is now reduced to a triangular shaped missing cone. The map used for this illustration is the same one shown in Figure 4.
